# Transcriptome analysis uncovers the autophagy‐mediated regulatory patterns of the immune microenvironment in dilated cardiomyopathy

**DOI:** 10.1111/jcmm.17455

**Published:** 2022-06-26

**Authors:** Shuo Sun, Jiangting Lu, Chaojie Lai, Zhaojin Feng, Xia Sheng, Xianglan Liu, Yao Wang, Chengchen Huang, Zhida Shen, Qingbo Lv, Guosheng Fu, Min Shang

**Affiliations:** ^1^ Department of Cardiology, Sir Run Run Shaw Hospital, School of Medicine Zhejiang University Hangzhou China; ^2^ Key Laboratory of Cardiovascular Intervention and Regenerative Medicine of Zhejiang Province Hangzhou China

**Keywords:** autophagy, dilated cardiomyopathy (DCM), immune microenvironment, immunity, transcriptome

## Abstract

The relationship between autophagy and immunity has been well studied. However, little is known about the role of autophagy in the immune microenvironment during the progression of dilated cardiomyopathy (DCM). Therefore, this study aims to uncover the effect of autophagy on the immune microenvironment in the context of DCM. By investigating the autophagy gene expression differences between healthy donors and DCM samples, 23 dysregulated autophagy genes were identified. Using a series of bioinformatics methods, 13 DCM‐related autophagy genes were screened and used to construct a risk prediction model, which can well distinguish DCM and healthy samples. Then, the connections between autophagy and immune responses including infiltrated immunocytes, immune reaction gene‐sets and human leukocyte antigen (HLA) genes were systematically evaluated. In addition, two autophagy‐mediated expression patterns in DCM were determined via the unsupervised consensus clustering analysis, and the immune characteristics of different patterns were revealed. In conclusion, our study revealed the strong effect of autophagy on the DCM immune microenvironment and provided new insights to understand the pathogenesis and treatment of DCM.

## 1 INTRODUCTION

Dilated cardiomyopathy (DCM) is a progressive heart muscle disease with left ventricular (LV) or biventricular dilatation and systolic dysfunction.^1^ Structural or functional abnormalities of the myocardium accompanied with DCM potentially lead to life‐threatening events such as arrhythmias, heart failure (HF) and sudden cardiac death.^2^ Decades of research have revealed multiple causes of DCM, including genetic mutations, infections, inflammations, autoimmune diseases, exposure to toxins and endocrine or neuromuscular causes.^1^ Due to different causes, DCM is usually classified as familial (genetic) or nonfamilial (nongenetic) forms. Around 15–30% DCM patients were diagnosed as familial DCM.^3^, ^4^ In the past 20 years, with the help of next‐generation sequencing, several mutations have been identified as DCM marker genes,^5^, ^6^, ^7^ which are *LMNA44*, *MYH7*, *TNNT2*, *TTN*, *RBM20*, *BAG3* and so on.^8^, ^9^, ^10^, ^11^, ^12^ Among them, TTN truncation mutation is the most common cause of DCM, occurring in ~25% of familial DCM cases and 18% of sporadic cases.^7^, ^13^ Although genetic prediction based on gene mutation is becoming a useful tool in clinic, there are limited reports on DCM diagnosis based on gene expression.

Besides genetic mutations, immune microenvironment changes are also leading causes for DCM.^7^, ^14^ Myocardial damage caused by DCM leads to inflammation with recruited immune cells into heart to repair damaged myocardium. Pathological examination of myocardial biopsy samples (or autopsy) from DCM patients often reveals evidence of activated inflammatory cell infiltration with gene expression patterns compatible with activated immune cells.^1^, ^15^ Moreover, cardiac‐specific autoantibodies can be detected from ~60% DCM patients and their relatives, which directly affect cardiomyocyte function and disease prognosis.^16^, ^17^, ^18^ In patients, inflammatory DCM may be familial and related to HLA antigens.^19^ For instance, cardiac infiltrated immune cells with abnormal class II HLA expression occupied approximately 50% among all biopsy samples.^20^ Therefore, determining the proportion and type of infiltrated immune cells and HLA genes expression pattern is crucial to establish the best treatment plan.

Among the entire immune system, autophagy is essential for cell development, function and homeostasis.^21^, ^22^ Cell‐autonomous inflammation is one of the key contributions of autophagy to the immunity.^23^, ^24^ Furthermore, it is also proved that autophagic activation participates in innate immunity response by mediating foreign pathogens clearance.^25^ By affecting cell metabolism, cytoplasmic quality and tissue homeostasis, autophagy has various connections with many human diseases.^26^, ^27^, ^28^ Recent years, autophagy has emerged as a major regulator of cardiac homeostasis. Autophagy preserves cardiac structure and function in normal conditions and is activated in a stress response, which will limit damage in both physiological and pathological conditions.^29^, ^30^ There are some studies focusing on the effects of autophagy on DCM. For example, Gil‐Cayuela et al. analysed the autophagy‐related gene expression changes in DCM patients by RNA‐seq.^31^ They found that in DCM patients, the expression changes of NRBP2 and CALCOCO2 were related to LV dysfunction and remodelling. Furthermore, primary fibroblasts from severe autosomal recessive DCM patients with mutations in PLEKHM2 gene exhibited abnormal endosomal subcellular distribution, abnormal lysosomal localization, and impaired autophagic flux marked by RAB5, RAB7 and RAB9 respectively.^32^ In addition, the combination of overactivated AKT–mTOR pathway and defective autophagy has been described in a mouse model of DCM carrying LMNA mutations.^33^ This study showed that intraperitoneal injection of temsirolimus, a derivative of the mTOR inhibitor rapamycin (sirolimus), in mice could reduce cardiac dilatation and enhance cardiac function. The authors demonstrated temsirolimus treatment decreased mTORC1 signalling, increased LC3‐II and decreased p62 protein level. Although all these studies have shown that there is a link between autophagy activity and DCM disease progression, this only explains the tip of the iceberg of autophagy's impact on DCM. The mechanism of how a large number of autophagy genes affect the immune microenvironment to regulate DCM remains to be explored.

Here, we systematically evaluated the effect of autophagy on DCM immune microenvironment. The risk prediction model based on the expression of 13 DCM‐related autophagy genes could effectively distinguish healthy and DCM samples. Next, to explore the connection between immune microenvironment and DCM, immunocyte infiltration, immune responses and HLA status in DCM were investigated, which showed strong correlation with autophagy. To further study how autophagy regulates DCM, the unsupervised clustering on the autophagy expression profile in DCM samples was performed, and two autophagy‐mediated regulation patterns in DCM were determined. These two subtypes showed distinct characteristics of immune microenvironment. Gene set variation analysis (GSVA) and the functional enrichment analysis indicated that the two autophagy‐mediated patterns are mainly respectively responsible for disease and metabolism related pathways. In addition, through weighted gene co‐expression network analysis (WGCNA), a co‐expression module (blue module) which strongly correlated with autophagy subtypes was identified. The findings provide a comprehensive overview about how autophagy regulates DCM through the immune microenvironment.

## 2 MATERIALS AND METHODS

### 2.1 Data preprocess

The data used in this study were RNA sequencing data of the LV from 166 healthy donors and 166 DCM samples from The Myocardial Applied Genomics Network (MAGNet; www.med.upenn.edu/magnet). The LV free‐wall tissues were harvested at the time of cardiac surgery from subjects with heart failure undergoing transplantation and from unused donor hearts with apparently normal function. The expression matrix was reserved in the gene expression omnibus (GEO) database (https://www.ncbi.nlm.nih.gov/geo/query/acc.cgi?acc=GSE141910),^34^ and the clinical characteristics of involved samples in GSE141910 were listed in Table S1. In addition, the validation of autophagy model was performed in another microarray dataset GSE57338 (https://www.ncbi.nlm.nih.gov/geo/query/acc.cgi?acc=GSE57338).^35^ The gene expression was detected by Affymetrix Human Gene 1.1 ST Array microarray. Gene probes were annotated as gene symbols. Probes without matching gene symbols and matching multiple symbols were excluded. Gene expression value of duplicate gene symbol was calculated as the median value.

### 2.2 Alteration analysis of autophagy between DCM and healthy samples

The differential analysis of autophagy genes between healthy and DCM samples was carried out using R package ‘limma’,^36^ and differentially expressed autophagy genes were identified with adjusted *p*‐value <0.01 and |log2FC| > 0.5. The protein–protein interaction network was constructed via STRING database (https://string‐db.org/).^37^ The DCM‐related autophagy genes were identified by univariate logistic regression with the cut‐off criteria of *p* value <0.0001. Then least absolute shrinkage and selection operator (LASSO) regression was performed to select the most useful biomarkers among DCM‐related autophagy genes and the diagnostic model with nonzero coefficients was constructed using the R package ‘glmnet’. Risk scores (RS) were calculated based on the regression coefficient of DCM‐related autophagy genes, defined as the risk of suffering from DCM:

Risk‐score = BCL2L1*(−1.7447) + BID*(−0.4268) +CALCOCO2*(−3.4052) + CASP1*0.3661 + CCL2*(−0.7031) + CX3CL1*1.0324 + CXCR4*0.2951 + EIF4EBP1*(−0.8020) + GRID1*0.0424 + NAMPT*(−1.4451) + NLRC4*(−0.4771) + NRG1*0.3089 + TP63*0.2200.

Principal components analysis (PCA) was used to reduce the number of dimensions to find similarities and differences of RS between healthy and DCM samples. The ROC curve was plotted to assess the classification performance of the classifier.

### 2.3 Validation of DCM‐related autophagy genes using Real‐Time Quantitative PCR (RT‐qPCR)

Serum samples were lyzed in QIAzol Lysis Reagent and miRNeasy Serum/Plasma Kit(QIAGEN)and quantified using Nanodrop 2000 software (Nanodrop Products). For mRNA quantification, cDNA was synthesized using the PrimeScript RT reagent kit (Takara,). RT‐qPCR was performed using PCR 218 reaction mixture (11203ES08,) on a LightCycler 480II system (Roche). Each sample was performed in six biological and three technical replications. The relative transcript levels were calculated using the 2^−ΔΔCT^ method, and 18S rRNA was used as the internal control to normalize the same samples. Primers are listed in Table S2. This study was approved by the Institutional Review Board of Sir Run Run Shaw Hospital, School of Medicine Zhejiang University (research 20,220,215–38). Informed consent was obtained from all the participants.

### 2.4 Correlation analysis between immune microenvironment and autophagy

Immune cell infiltration was calculated using CIBERSORT algorithm based on the expression matrix of 22 types of immunocytes.^38^ The relative enrichment of the activity of immune pathways was determined by single‐sample gene set enrichment analysis (ssGSEA). The immune reaction gene sets used to evaluate the activity of immune‐related pathways were obtained from ImmPort (http://www.immport.org).^39^ The relative fraction of immunocytes, enrichment score of immune‐related pathways and expression of HLA genes between healthy and DCM samples were compared using the Wilcox test. The correlation analysis between the relative fraction of immunocytes, enrichment score of immune pathways and expression of HLA genes and autophagy genes were done by Spearman correlation analysis with R package ‘corrplot’.

### 2.5 Identification of autophagy expression patterns

Unsupervised clustering analysis was performed to identify distinct autophagy expression patterns according to the expression of all 201 autophagy genes. Consensus clustering was implemented to evaluate the autophagy expression pattern using the R package ‘ConsensuClusterPlus’.^40^ This algorithm was repeated 1000 times to ensure the stability of classification. The expression of the DCM‐related autophagy genes, immunocyte relative fraction, immune reaction scores and expression of HLA genes between the two subtypes were compared using the Wilcox test.

### 2.6 Functional enrichment analysis of the two autophagy expression patterns

To screen for autophagy expression pattern related genes, differentially expressed genes between two subtypes were identified using the R package ‘limma’.^36^ The criterion for DEGs was |log2FC| > 0.5 and adjusted *p*‐value <0.001. The biological characteristics of autophagy phenotype‐related genes and were uncovered by GO and KEGG enrichment analysis using the R package ‘clusterProfiler’.^41^

To further reflect biological changes that occurred in each subtype, the gene‐sets of ‘h.all.v7.0.symbols’ and ‘c2.cp.kegg.v7.0.symbols’ were downloaded from MSigDB (http://www.gsea‐msigdb.org/).^42^ Next, the activity of HALLMARKS and KEGG pathway was quantified using the GSVA algorithm,^43^ and was compared between two subtypes using the Wilcox test. Pathways with adjusted *p*‐value <0.01 were considered to be significantly different in activity between the two subtypes.

### 2.7 Identification of autophagy expression pattern related gene modules

Prior to analyse co‐expressed genes, the coefficient of variation (CV) of each gene based on their expression values were calculated and genes with low variability (CV <0.1) were filtered. Next, the R package WGCNA was used to identify modules of highly correlated genes across all samples.^44^ The parameters of WGCNA used default settings, except for the power was 2, network type was signed, minModuleSize was 30. Eigengenes and clusters were calculated based on the correlations to quantify the co‐expression similarity of entire modules, using a strict cut‐off of 0.25, corresponding to correlation of 0.75. Then, kME, known as the module membership value, was calculated using SignedKME algorithm to represent the correlation between a gene and the module eigengene value. Intramodular hub genes of module blue were identified if their kME >0.95.

## 3 RESULTS

### 3.1 The landscape of autophagy gene alterations in DCM

**FIGURE 1:**
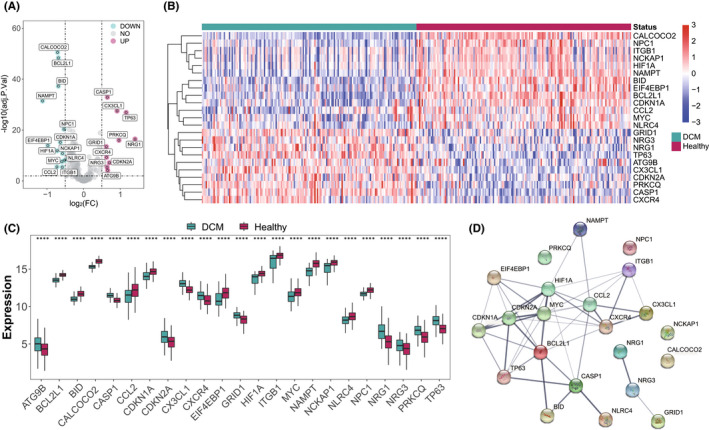
Expression landscape autophagy genes in DCM. (A) The volcano‐plot shows the summary of expression changes of 201 autophagy genes between healthy and DCM samples and the 23 significant dysregulated autophagy genes are labelled. (B,C) The heatmap‐plot and box‐plot demonstrate the expression pattern of 23 significantly dysregulated autophagy genes between healthy and DCM samples. (D) The 23 significant dysregulated autophagy gene protein–protein interaction network

To explore the expression status in DCM, 222 known autophagy genes were collected from Human Autophagy Database (http://www.autophagy.lu/). Here, a set of RNA‐seq data sampled from the LV of 166 healthy donors and 166 DCM patients was used for analysis, and the expression of 201 autophagy genes in all samples were obtained. The differential analysis revealed that there were 23 significantly dysregulated autophagy genes, 10 of which were significantly induced in DCM samples, while the rest were significantly suppressed (Figure 1A‐C, Table S3). In particular, NAMPT had the largest fold change and CALCOCO2 had the most statistically significant change. To reveal the interactions between these autophagy genes, the protein–protein interaction network was constructed (Figure 1D). Among them, HIF1A and BCL2L1 interacted with more than 10 autophagy genes.

### 3.2 Autophagy genes can well distinguish healthy and DCM samples

**FIGURE 2:**
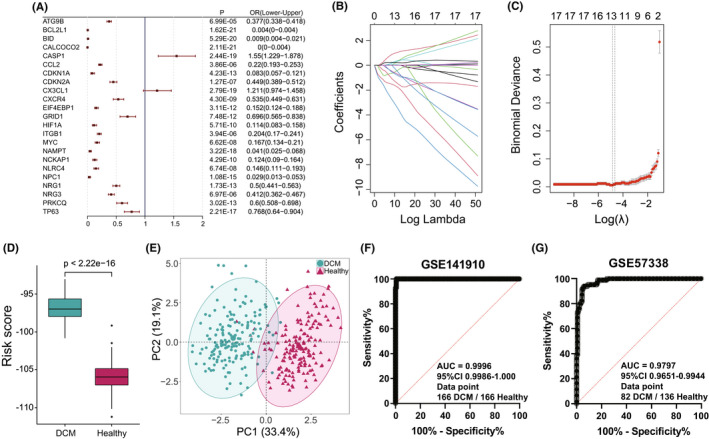
Autophagy genes can distinguish healthy and DCM samples. (A) Univariate logistic regression investigates the relationship between dysregulated autophagy genes and DCM. (B) LASSO coefficient profiles of 23 DCM‐related autophagy genes. (C) Ten‐fold cross‐validation for tuning parameter selection in the LASSO regression. The partial likelihood deviance is plotted against log (λ), where λ is the tuning parameter. Partial likelihood deviance values are shown, with error bars representing SE. The dotted vertical lines are drawn at the optimal values by minimum criteria and 1‐SE criteria. (D) The box‐plot compares the RS obtained by the LASSO regression model between healthy and DCM samples, where DCM has a much higher RS than healthy samples. (E) Principal component analysis (PCA) of 10 DCM‐related autophagy genes between healthy and DCM samples. (F,G) ROC curves and AUC values evaluate the discrimination ability for healthy and DCM samples by autophagy genes in the training and validation set

To further verify crucial autophagy genes in DCM, a series of bioinformatic algorithms were applied to the 23 significantly altered autophagy genes. First, univariate logistic regression was used to identify DCM‐related autophagy genes, and all 23 autophagy genes were verified closely related to DCM (Figure 2A). Next, LASSO regression was performed for feature selection and dimension reduction to exclude redundant autophagy genes, and 13 DCM‐related autophagy genes were found (Figure 2B,C). To develop a classifier to distinguish healthy and DCM samples, the prediction model was constructed based on the LASSO regression coefficient of DCM‐related autophagy genes and the RS of each sample was obtained (Figure 2D, Table S4). As expected, the overall RS of the DCM samples were significantly higher than those of the healthy samples (*p* < 2.22e‐16). Since the RS of the 365/366 healthy samples were less than −100, while 363/366 DCM samples were greater than −100, −100 could be regarded as the risk threshold for distinguishing healthy and DCM samples (Table S5). Furthermore, principal component analysis (PCA) demonstrated that there were diverse autophagy gene expression patterns between healthy and DCM samples (Figure 2E). In addition, the receiver operating characteristic (ROC) curve also showed that this autophagy model achieved an AUC value of 0.996 in classifying healthy and DCM (Figure 2F). Given that the result was based on the model training dataset, another dataset including 82 DCM samples and 136 healthy samples was selected to further validate this model. The validation generated a similar result (AUC = 0.9797), which indicated the robustness of the model (Figure 2G). In addition, in order to clarify the spatiotemporal regulation of autophagy in DCM in the human body, peripheral serum samples from 6 DCM patients and 6 healthy people were collected. RT‐qPCR results showed that the expression trend of 6/13 DCM‐related autophagy genes (BCL2L1, BID, CALCOCO2, NAMPT, EIF4EBP1, CCL2) in peripheral serum were opposite to those in LV (Figure S1), which also indicated that the risk prediction model may not be suitable for peripheral serum samples.

### 3.3 Autophagy is associated with immune microenvironment in DCM

**FIGURE 3:**
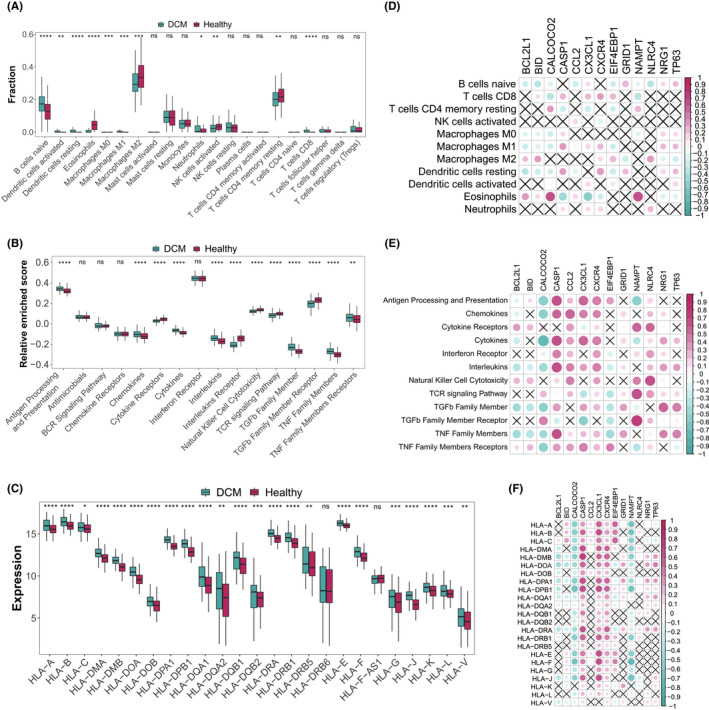
The correlation between immune microenvironment characteristics (infiltrating immunocytes, immune reaction gene‐sets, HLA genes) and autophagy genes. (A‐C) The difference in the abundance of each infiltrating immunocyte, immune reaction gene‐set and HLA gene between healthy and DCM samples. (D‐F) The dot‐plot demonstrate the correlations between each dysregulated infiltrating immunocyte, immune reaction gene‐set and HLA gene, and each dysregulated autophagy gene

To further investigate the biological connections between autophagy genes and immune microenvironment, the correlation analysis for dysregulated autophagy genes with infiltrating immunocytes, immune reaction gene‐sets and HLA gene expression in DCM was performed. Immune infiltration analysis showed that dramatic changes in 11 of 22 immunocytes occurred in DCM samples (*p* < 0.05). Most of the dysregulated immunocytes were upregulated in DCM such as B cells naive, Dendritic cells activated and T cells CD8 (Figure 3A, Table S6), suggesting a great change of immune microenvironment during DCM progression. Correlation analysis showed autophagy genes were closely related to many immunocytes (Figure 3D, Table S7). The significant positively correlated immunocyte‐autophagy gene pair is CALCOCO2‐eosinophils (Figure S2A), while the most positively correlated is NAMPT‐eosinophils (Figure S2B). In contrast, the most negatively correlated pair is CX3CL1‐eosinophils (Figure S2C).

Likewise, the activity of immune‐related pathways and expression levels of HLA genes were calculated and obvious changes were observed between healthy and DCM samples (Figures 3B,C). Interestingly, nearly all HLA genes were highly expressed in DCM samples (*p* < 0.05). Their correlations with autophagy were also fully revealed (Figures 3E,F, Table S8–S11). For immune‐related pathways, the most positively correlated pair is NAMPT‐TGFb Family Member Receptor (Figure S3A); while the most negatively correlated pair is CALCOCO2‐cytokines (Figure S3B). For HLA genes, the most positively correlated HLA‐autophagy pair is CASP1‐HLA‐DMB (Figure S4A); while the most negatively correlated pair is NAMPT‐HLA‐F (Figure S4B). It is worth mentioning that CASP1 and CXCR4 were significantly positively correlated with all dysregulated HLA genes, which indicated that these two autophagy genes were closely related to HLA in DCM.

### 3.4 Two autophagy mediated modification patterns and their relationship to the immune microenvironment in DCM

**FIGURE 4:**
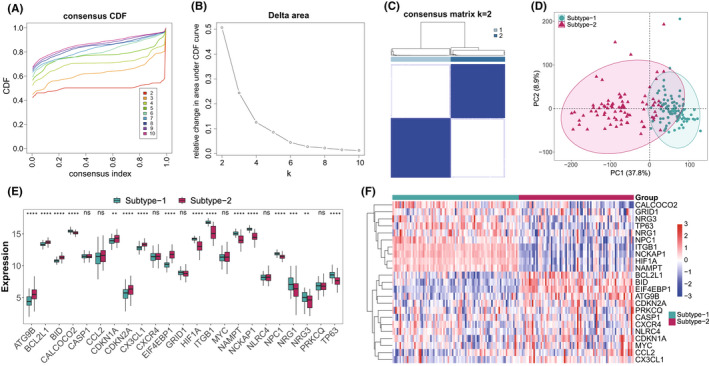
Unsupervised clustering of 201 autophagy genes identifying 2 distinct autophagy‐mediated regulation pattern subtypes in DCM. (A) Consensus clustering cumulative distribution function (CDF) for k = 2–10. (B) Relative change in area under the CDF curve for k = 2–10. (C) Heatmap of the matrix of co‐occurrence proportions for DCM samples. (D) Principal component analysis for the transcriptome profiles of 2 autophagy regulation patterns, showing a clear distinction in transcriptome between different regulation patterns. (E,F) The box‐plot and heatmap‐plot demonstrate the expression pattern of 23 significantly dysregulated autophagy genes between 2 autophagy regulation patterns

To explore the autophagy mediated patterns in DCM, the unsupervised consensus clustering analysis was conducted on DCM samples based on the expression of 201 autophagy genes (Figure 4A–C). Two distinct subtypes of DCM were identified with qualitatively different expression of 201 autophagy genes, including 87 samples in subtype‐1 and 79 samples in subtype‐2 (Table S12). PCA analysis revealed that there was a remarkable difference in transcriptome between the two subtypes (Figure 4D). Then, the expression of the 23 significantly dysregulated autophagy genes were compared between two DCM subtypes, and only 16 of them dramatically altered (Figure 4E,F). This indicated that these 16 autophagy genes may be involved in different mechanisms of regulating DCM, while the rest may regulate DCM indiscriminately.

**FIGURE 5:**
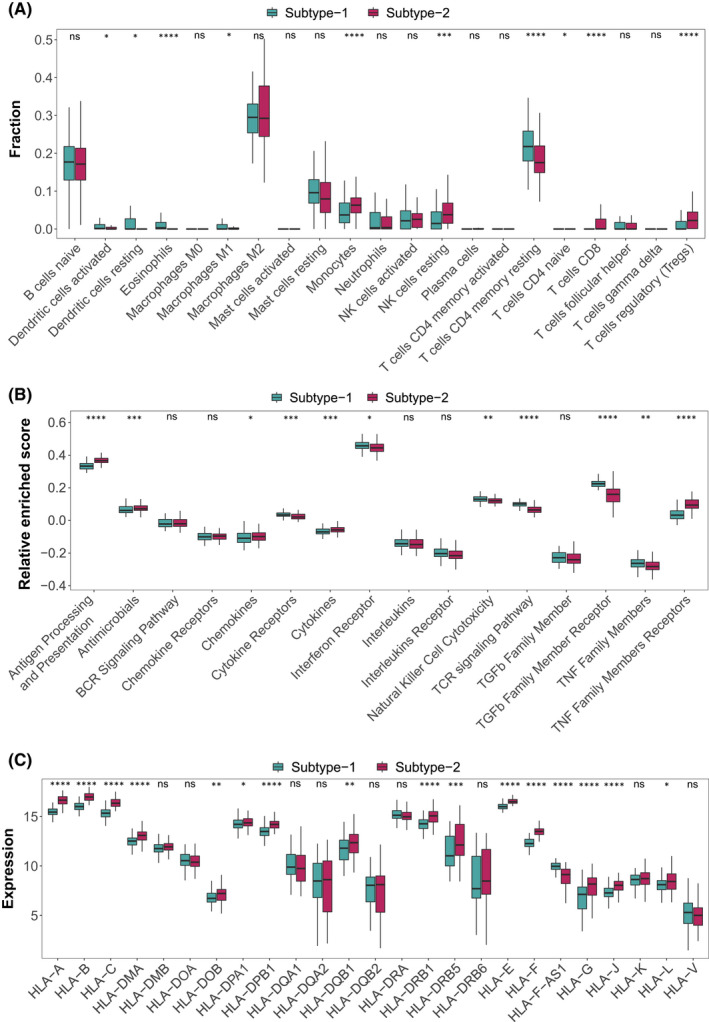
Diversity of immune microenvironment characteristics between distinct autophagy‐mediated regulation patterns. (A) The abundance differences of infiltrating immunocytes between 2 autophagy regulation patterns. (B) The activity differences of immune reaction gene‐sets between 2 autophagy regulation patterns. (C) The expression differences of HLA genes between 2 autophagy regulation patterns

To uncover the differences of immune microenvironment characteristics between the two subtypes, infiltrating immunocytes, immune response gene‐sets and HLA gene expression were evaluated. As expected, the two subtypes demonstrated very distinct autophagy‐mediated immune characteristic (Figure 5, Table S13). For example, much more eosinophils and resting memory CD4 T cells were observed in subtype‐1, while higher levels of Monocytes, CD8 T cells and regulatory T cells (Tregs) were found in subtype‐2 (*p* < 0.0001). As for immune responses, TCR signalling pathway and TGF family member receptor related genes were enriched more in subtype‐1, while antigen processing and presentation and TNF family members receptors were more active in subtype‐2 (*p* < 0.0001). In particular, with the exception of HLA‐F‐AS1, all dysregulated HLA genes had higher expression levels in subtype‐2, suggesting subtype‐2 represents immune enrichment.

### 3.5 Biological functions behind autophagy expression patterns

**FIGURE 6:**
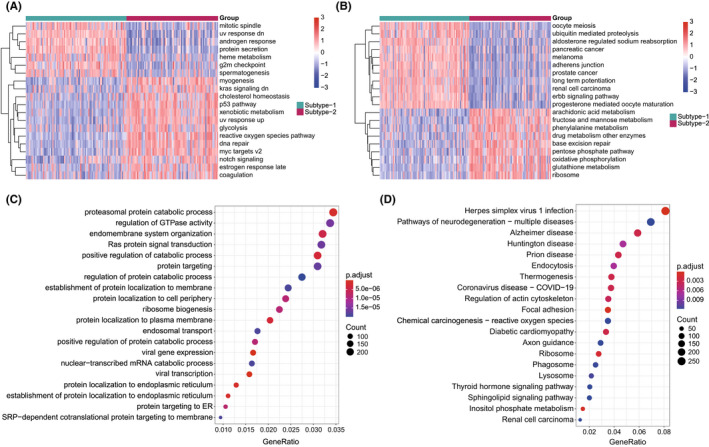
The underlying biological characteristics diversity between 2 autophagy‐mediated regulation patterns. (A,B) The top 20 HALLMARKS and KEGG pathways with the most significant differences between 2 autophagy regulation patterns (A for HALLMARKS pathway and B for KEGG pathway). (C, D) GO‐BP functional and KEGG enrichment analysis for autophagy phenotype‐related genes (C for GO enrichment and D for KEGG enrichment)

Differences in expression and immune microenvironment characteristics illustrated that there are two mechanisms for autophagy genes to regulate DCM. In order to clarify the respective roles of the two subtypes, GSVA analysis was employed to calculate the enrichment scores of HALLMARK and KEGG pathways. Interestingly, some significantly enriched HALLMARK pathways that may be directly related to the dilation of the heart of DCM patients were detected. For example, heme metabolism was highly enriched in subtype‐1, and myogenesis was enriched in subtype‐2 (Figure 6A). Besides, several cancer related KEGG pathways were dramatically enriched in subtype‐1, such as renal cell carcinoma, pancreatic cancer, prostate cancer and melanoma (Figure 6B). In contrast, various metabolic pathways were more active in subtype‐2, including arachidonic acid metabolism, fructose and mannose metabolism, phenylalanine metabolism, glutathione metabolism and drug metabolism other enzymes. To further understand the role of autophagy in immunity, 9604 differentially expressed common genes (|log2FC| > 0.5, adjusted p‐value <0.001) between the two subtypes were identified as autophagy subtype‐related genes (Table S14). Gene ontology (GO)‐BP enrichment analysis revealed that they were mainly involved in proteasomal protein catabolic process, regulation of GTPase activity and endomembrane system organization (Figure 6C). Furthermore, KEGG enrichment result also demonstrated the autophagy subtype‐related genes were mostly enriched in multiple disease related pathways, such as Alzheimer disease, Huntington disease, Prion disease and Coronavirus disease‐COVID‐19 (Figure 6D). This was consistent with the results of GSVA analysis.

### 3.6 Co‐expression network analysis identified autophagy expression pattern related gene modules

**FIGURE 7:**
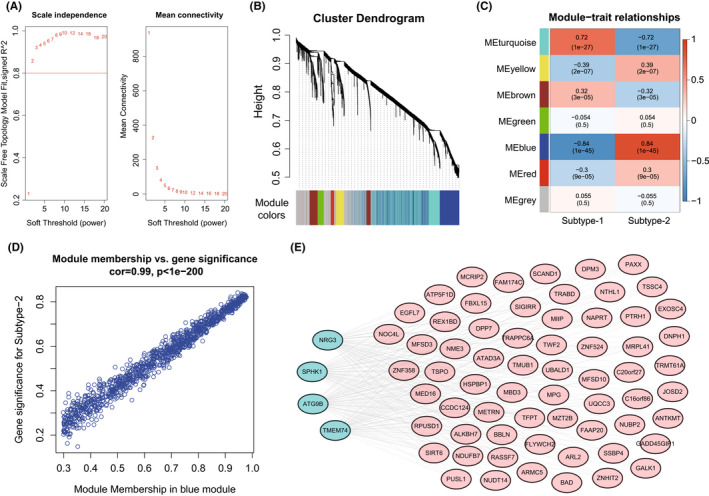
Co‐expression gene modules related to autophagy‐mediated patterns. (A) Analysis of the scale‐free fit index and analysis of the mean connectivity for various soft‐thresholding powers. (B) Gene dendrogram obtained by average linkage hierarchical clustering. The colour row underneath the dendrogram shows the module assignment determined by the Dynamic Tree Cut, in which 7 modules were identified. (C) Heatmap of the correlation between module eigengenes and the autophagy‐mediated regulation subtypes. (D) A scatterplot of gene significance (GS) for autophagy‐mediated subtype‐2 versus module membership (MM) in the blue module. GS and MM exhibit a very significant correlation, implying that hub genes of the blue module also tend to be highly correlated with autophagy‐mediated regulation subtype‐2. (E) The interaction network of DCM‐related autophagy genes and hub genes in the blue module

Next, a comprehensive gene landscape correlated to each autophagy expression patterns was constructed, and gene co‐expression modules related to distinct autophagy regulations were identified by weighted gene co‐expression network analysis (WGCNA) (Figure 7A,B). Seven gene modules were determined and different expression pattern matched their related genes (Figure 7C, Table S15). Among them, the most relevant to the autophagy subtype was the blue module (Figure 7D), which was closely correlated to the subtype‐2 (cor = 0.99, *p* < 1e‐200). In order to further figure out the regulatory roles of autophagy genes in the blue module, the interaction network including DCM‐related autophagy genes and the hub genes (kME >0.95) in this module was explored. As a result, the interaction between four autophagy genes (NRG3, SPHK1, ATG9B, TMEM74) and 64 hub genes was discovered (Figure 7E). Overall, these results could shed light on the gene expression regulation network mediated by autophagy.

## 4 DISCUSSION

Autophagy is a bridge connecting innate immunity and adaptive immunity.^21^, ^45^ For a long time, it has been considered to play an important role in antigen presentation, maintenance of lymphocyte homeostasis and regulation of cytokine production.^23^ However, there are few reports on how autophagy shapes the immune microenvironment of DCM. Knowing that autophagy is essential for immune response, we believe that autophagy must have a significant impact on the shaping of the immune microenvironment of DCM. The dataset GSE141910 used in this study includes 200 HF samples and 166 non HF samples. The HF samples consist peripartum cardiomyopathy, hypertrophic cardiomyopathy and DCM samples. Although the publication of GSE141910 has not yet been released, two studies have conducted a series of bioinformatics analyses using this dataset. These two studies were focused on evaluating the significance of immune infiltration in the pathogenesis of hypertrophic cardiomyopathy,^34^ and screening hub genes involved in developmental HF as well as to explore active drug molecules,^46^ respectively. Through reanalysis of this dataset, this study systematically evaluated the relationship between autophagy and immune microenvironment (immune cells, immune responses, HLA genes) in DCM (Figure 3). In this way, we could find out some novel connections between autophagy and immune microenvironment changes, and enrich the understanding of the reported relationships. For instance, CALCOCO2 and NAMPT, two autophagy genes known to be associated with LV dysfunction,^31^, ^47^ were found to be significantly positively correlated with Eosinophils in this study. This suggested that the two autophagy genes may regulate DCM by affecting the infiltration of Eosinophils. These findings could play a very enlightening role in the development of immunotherapy from the perspective of autophagy in DCM.

The heterogeneous aetiology and clinical characteristics of DCM make a correct and timely diagnosis challenging.^1^, ^3^ At present, echocardiography and other imaging techniques are needed to assess ventricular dysfunction and poor myocardial remodelling. When inflammation or infection is suspected, immunological and histological analysis of endocardial myocardial biopsy samples are also required, which makes the diagnosis of DCM in the clinic too cumbersome. Recent years, although gene sequencing technology based on the gene mutation analysis has been used for the diagnosis of DCM,^5^, ^7^, ^48^ this relies heavily on the detailed information of family genetic history. Besides, the accuracy of this approach needs to be improved. With the outbreak of COVID‐19, the nucleic acid detection method has become the most popular genetic diagnosis technology. In this study, 13 DCM‐related autophagy genes were screened through a series of bioinformatics methods and a risk diagnosis model was constructed, which can distinguish healthy and DCM samples well (Figure 2). This model may play an important role in the diagnosis and treatment of DCM, but the effect on the diagnosis of early DCM needs to be further verified by more data.

Morever, we have identified two distinct autophagy expression patterns, which are different from any other classification for DCM. The autophagy expression patterns could help us deepen our understanding of autophagy in DCM and how it shapes the immune microenvironment. It is worth mentioning that a previous study identified 12 up‐regulated proteins between DCM and healthy samples by proteomics.^49^ Although autophagy genes were not included, three proteins (*GSTP1*, *SORBS2*, *MYBPC3*) were differentially expressed between the two autophagy subtypes, suggesting that these three DCM up‐regulated proteins may be involved in autophagy regulation. Since a close correlation between autophagy and immune microenvironment was detected in DCM, we wonder if different autophagy subtypes would show different immune characteristics. The results proved our point, that these two subtypes were very different in terms of immunocyte composition, immune responses and HLA gene expression. Additionally, to further clarify the regulation mechanism of autophagy on DCM, GSVA was performed on different autophagy subtypes, and the results may provide ideas to explain some DCM clinical characteristics (Figure 6). For example, men generally have a higher risk for DCM than women.^1^ It has been reported that sex hormones can change cardiac function by binding to androgen and oestrogen receptors on cardiac vascular endothelial cells, smooth muscle cells, fibroblasts and muscle cells.^50^ In addition, the binding of sex hormones to their receptors directly changes the functions of immune cells and platelets, thereby affecting the type of cardiac inflammation, remodelling and thrombosis in DCM. Interestingly, the androgen response pathway was highly enriched in autophagy subtype‐1, while the oestrogen response pathway was highly enriched in subtype‐2. This indicated that these two distinct autophagy‐mediated expression patterns respond to different sex hormones, which may lead to gender differences in the risk of DCM. In addition, some cancer‐related pathways were highly enriched in subtype‐1, and the KEGG enrichment analysis of the differential expressed genes between the two subtypes also showed that the most significantly enriched pathways were related to diseases. This suggested that the regulation of autophagy may be involved in the complications of DCM and other diseases.

Overall, our findings revealed how autophagy affects the immune microenvironment of DCM, and provided new insights to understand the pathogenesis of DCM. This study was the first to systematically reveal the potential connections between autophagy and immune microenvironment in DCM. These findings provide clues for further studying the mechanism of autophagy in DCM.

## AUTHOR CONTRIBUTIONS

**Shuo Sun:** Conceptualization (lead); formal analysis (lead); writing – original draft (lead); writing – review and editing (lead). **Jiangting Lu:** Investigation (equal); validation (lead). **Chaojie Lai:** Validation (supporting). **Zhaojin Feng:** Investigation (equal). **Sheng Xia:** Investigation (equal). **Lan Xiang:** Investigation (equal). **Yao Wang:** Conceptualization (supporting). **Chengchen Huang:** Investigation (equal). **Zhida Shen:** Investigation (equal). **Qingbo Lv:** Investigation (equal). **Guosheng Fu:** Supervision (supporting). **Min Shang:** Conceptualization (supporting); supervision (supporting); writing – review and editing (supporting).

## CONFLICT OF INTEREST

The authors declare that they have no competing interests.

## Supporting information


Figure S1

Figure S2

Figure S3

Figure S4

Table S1

Table S2

Table S3

Table S4

Table S5

Table S6

Table S7

Table S8

Table S9

Table S10

Table S11

Table S12

Table S13

Table S14

Table S15

Data S1

## Data Availability

The data that support the findings of this study are available in [https://www.ncbi.nlm.nih.gov/geo/query/acc.cgi?acc=GSE141910] and [https://www.ncbi.nlm.nih.gov/geo/query/acc.cgi?acc=GSE57338].
