# Supplementary figures and images for: Transcriptome analysis uncovers the autophagy‐mediated regulatory patterns of the immune microenvironment in dilated cardiomyopathy
Source: J Cell Mol Med. 2022 Jun 26;26(14):4101–12. doi: 10.1111/jcmm.17455 (PMC9279601; doi:10.1111/jcmm.17455)

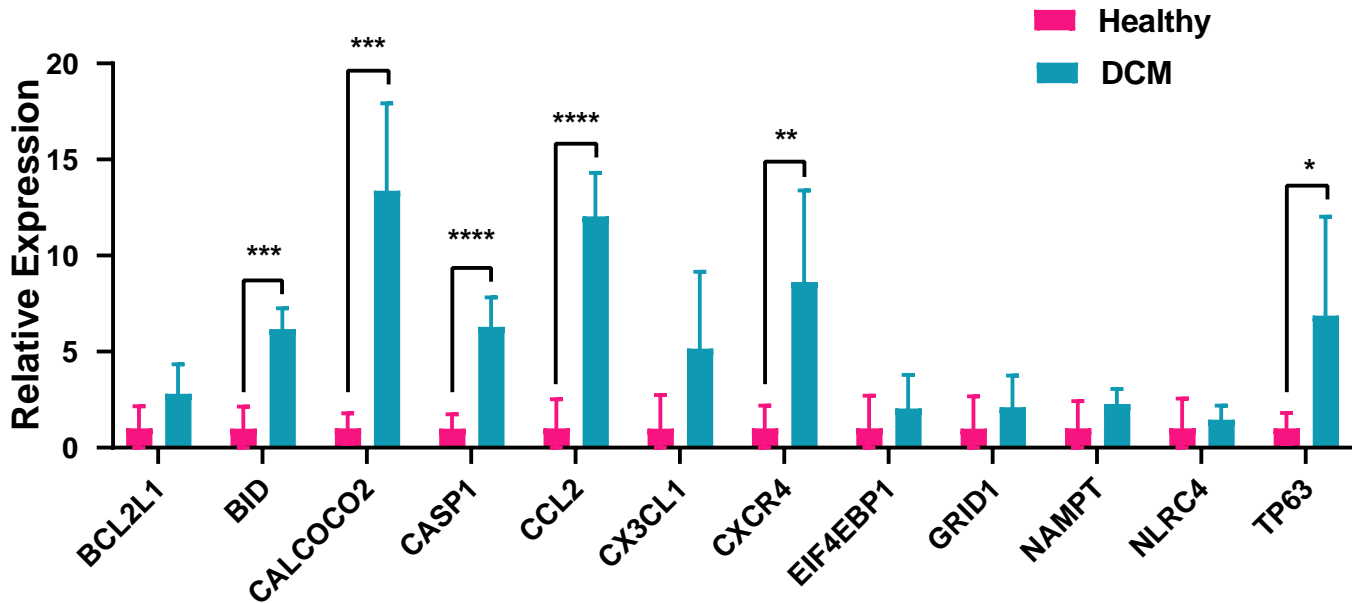

Supplement: Supplementary file 1 — Figure S1 [file JCMM-26-4101-s005.pdf]

A

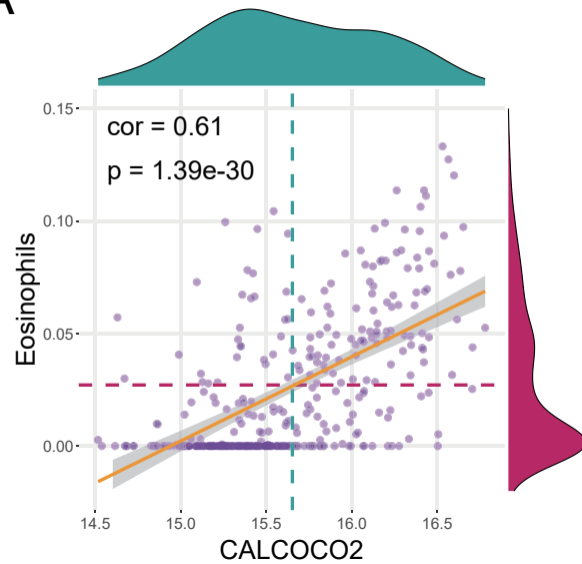

B

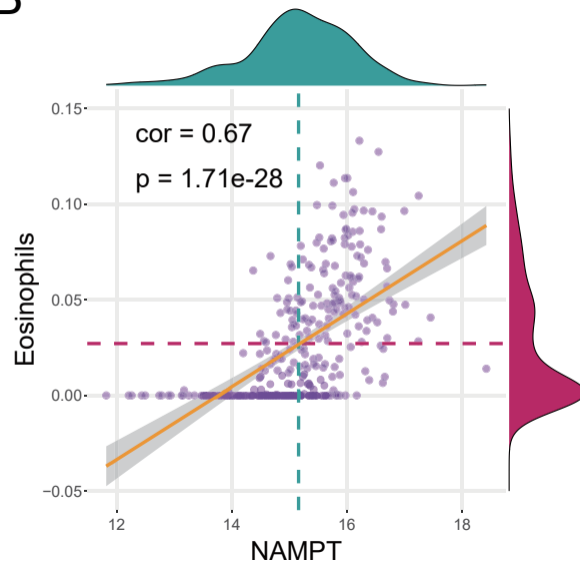

C

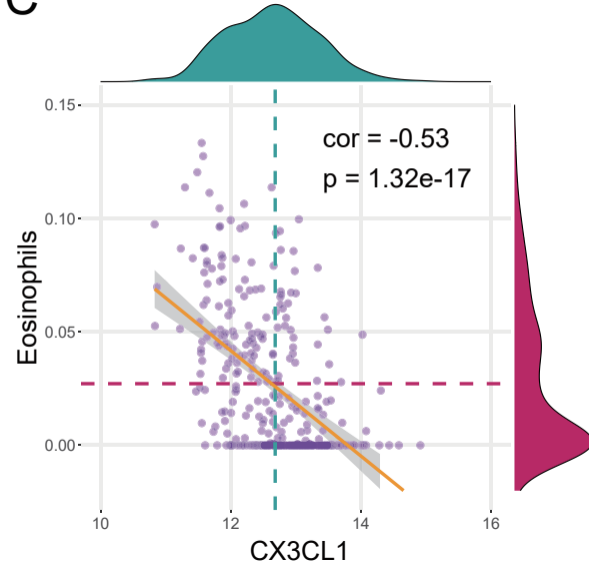

Supplement: Supplementary file 2 — Figure S2 [file JCMM-26-4101-s010.pdf]

A

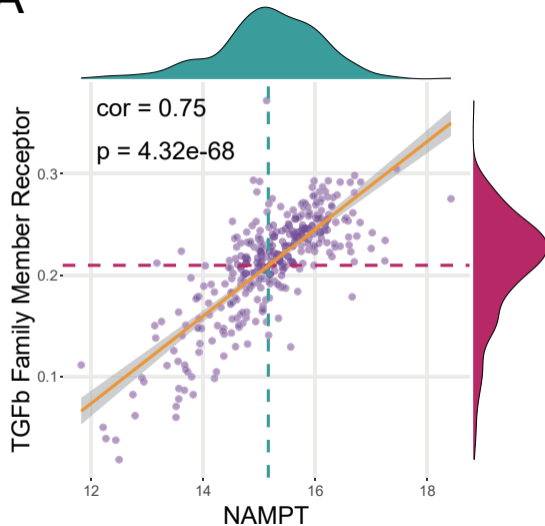

B

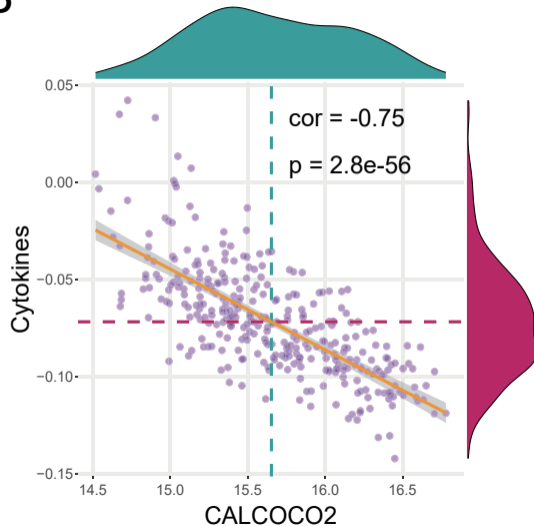

Supplement: Supplementary file 3 — Figure S3 [file JCMM-26-4101-s019.pdf]

**A**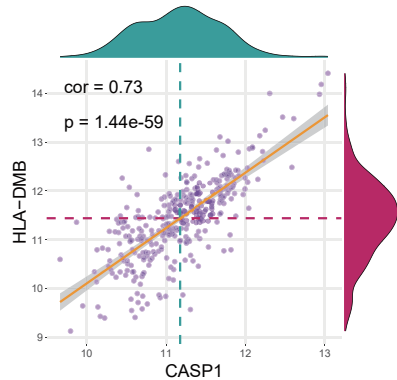**B**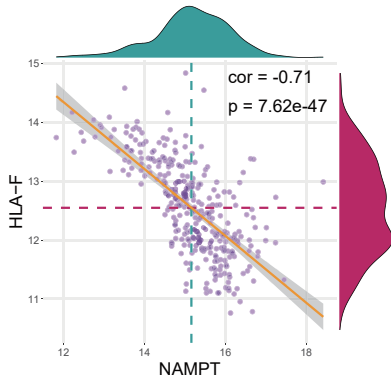

Supplement: Supplementary file 4 — Figure S4 [file JCMM-26-4101-s013.pdf]
