# Supplementary material for: Transcriptome analysis uncovers the autophagy‐mediated regulatory patterns of the immune microenvironment in dilated cardiomyopathy
Source: J Cell Mol Med. 2022 Jun 26;26(14):4101–12. doi: 10.1111/jcmm.17455 (PMC9279601; doi:10.1111/jcmm.17455)
Supplement: Supplementary file 20 — Data S1 [file JCMM-26-4101-s014.docx]

Figure S1 Relative expression of DCM-related autophagy genes in the peripheral serum of DCM patients and healthy donors. Vertical bars represent SD of the mean of three biological replicates. ^*^P < 0.05; ^**^P < 0.01; ^***^P < 0.001; ^****^P < 0.0001; one-way anova followed by Tukey test.

Figure S2 Scatter plots display the most significant positively, the most positively and the most positively correlated immunocyte-autophagy gene pairs are immunocyte-autophagy gene pairs are CALCOCO2-Eosinophils (A), NAMPT-Eosinophils (B) and CX3CL1-Eosinophils (C), respectively.

Figure S3 Scatter plots display the most positively and the most positively correlated immune reaction-autophagy gene pairs are NAMPT-TGFb Family Member Receptor (A) and CALCOCO2-Cytokines (B), respectively.

Figure S4 Scatter plots display the most positively correlated and the most negatively HLA-autophagy pairs are CASP1-HLA-DMB (A) and NAMPT-HLA-F (B), respectively.
